# High Resolution Mapping of Modafinil Induced Changes in Glutamate Level in Rat Brain

**DOI:** 10.1371/journal.pone.0103154

**Published:** 2014-07-28

**Authors:** Mohammad Haris, Anup Singh, Kejia Cai, Kavindra Nath, Gaurav Verma, Ravi Prakash Reddy Nanga, Hari Hariharan, John A. Detre, Neill Epperson, Ravinder Reddy

**Affiliations:** 1 CMROI, Radiology, University of Pennsylvania, Philadelphia, Pennsylvania, United States of America; 2 Research Branch, Sidra Medical and Research Center, Doha, Qatar; 3 Center for Biomedical Engineering, Indian Institute of Technology Delhi, Delhi, India; 4 CMRR, Radiology, University of Illinois at Chicago, Chicago, Illinois, United States of America; 5 Molecular Imaging, University of Pennsylvania, Philadelphia, Pennsylvania, United States of America; 6 Department of Neurology, University of Pennsylvania, Philadelphia, Pennsylvania, United States of America; 7 Psychiatry, University of Pennsylvania, Philadelphia, Pennsylvania, United States of America; Western University of Health Sciences, United States of America

## Abstract

Modafinil is marketed in the United States for the treatment of narcolepsy and daytime somnolence due to shift-work or sleep apnea. Investigations of this drug in the treatment of cocaine and nicotine dependence in addition to disorders of executive function are also underway. Modafinil has been known to increase glutamate levels in rat brain models. Proton magnetic resonance spectroscopy (^1^HMRS) has been commonly used to detect the glutamate (Glu) changes *in vivo*. In this study, we used a recently described glutamate chemical exchange saturation transfer (GluCEST) imaging technique to measure Modafinil induced regional Glu changes in rat brain and compared the results with Glu concentration measured by single voxel ^1^HMRS. No increases in either GluCEST maps or ^1^HMRS were observed after Modafinil injection over a period of 5 hours. However, a significant increase in GluCEST (19±4.4%) was observed 24 hours post Modafinil administration, which is consistent with results from previous biochemical studies. This change was not consistently seen with ^1^HMRS. GluCEST mapping allows regional cerebral Glu changes to be measured and may provide a useful clinical biomarker of Modafinil effects for the management of patients with sleep disorders and addiction.

## Introduction

Modafinil is an analeptic medication used clinically in the treatment of narcolepsy, shift work disorder, and daytime somnolence due to sleep apnea without interfering with nocturnal sleep [1]–[3]. It increases wakening in rodents (Simon *et al*, 1994; Touret *et al*, 1995), monkeys (Hermant *et al*, 1991; Lagarde and Milhaud, 1990) and humans [4]. It is now also undergoing clinical trials for the treatment of dependence on psycho-stimulants such as cocaine and nicotine [5]–[7] and attention deficit disorder with and without comorbid affective disorders [8]–[10]. In addition, protective effects of Modafinil have been observed in experimental hypoxia, ischemic injury and in a model of Parkinson's disease [11]–[14]. Although Modafinil's mechanism of action is not well characterized, it has been shown that Modafinil induced wakefulness is associated with an increase in cerebral glutamate (Glu) levels [15].

Magnetic resonance spectroscopy (MRS) is a non-invasive technique that has been widely used to detect Glu concentration *in vivo* [16], [17]. Although prior studies have been performed to monitor the Modafinil induced changes in brain Glu level, the data regarding the time course of Modafinil effect on Glu levels are not clear [15], [18]. A 2D COSY MRS study in rat brain has shown that Modafinil increased cerebral Glu level significantly within few hours (2–7 hours) [18]. In another study using high performance liquid chromatography (HPLC) observed an increase in Glu at 12 to 24 hours, but not at 2–7 hours, post Modafinil administration [15].

Recently, imaging of Glu *in vivo* in human and rat brains was performed at high spatial resolution using chemical exchange saturation transfer (GluCEST) imaging method [19]. Briefly, in GluCEST the labile amine protons of Glu can be selectively saturated with the application of radiofrequency pulse and their transfer with bulk water leads to a decrease in water signal in a concentration dependent manner. The application of GluCEST has been shown earlier in detecting the changes in brain Glu concentration in mouse model of Alzheimer's disease [20].

The aim of the current study was to image Modafinil induced regional Glu level changes in healthy rat brain, *in vivo*, using the GluCEST method and compare the findings with absolute glutamate/glutamine (Glx) concentration changes measured with^1^HMRS.

## Materials and Methods

The Institutional Animal Care and Use Committees of the University of Pennsylvania approved all the experimental protocols in this study. MR imaging was performed at 9.4T horizontal bore small animal MR scanner (Varian, Palo Alto, CA) using a 35-mm diameter commercial quadrature proton coil (m2m Imaging Corp., Cleveland, OH). All Modafinil solutions were prepared in 0.3% tragcanth gum solution as a suspension and injected peritoneally. A total of fifteen Sprague Dawley rats were used in this study split into 4 groups. For the group 1 (n = 5), Modafinil dose of 500 mg/kg was used and ^1^HMRS and GluCEST studies were performed pre Modafinil injection and every 30 min post Modafinil injection for a period of 5 hours. For the group 2 (n = 6), Modafinil dose of 500 mg/kg was used and ^1^HMRS and GluCEST studies were performed pre Modafinil injection and 24 hours post Modafinil injection. For the group 3 (n = 2), Modafinil dose of 200 mg/kg was used and ^1^HMRS and GluCEST studies were performed pre Modafinil injection and 24 hours post Modafinil injection. The group 4 animal (n = 2), did not get any Modafinil injection, but underwent multiple GluCEST imaging experiments to measure GluCEST reproducibility. During MR imaging and spectroscopy measurements, animals were kept under anesthesia (1.5% isoflurane in 1 liters/min oxygen) and their body temperature maintained with the air generated and blowing through a heater (SA Instruments, Inc., Stony Brook, NY).

### GluCEST MR Imaging

GluCEST imaging of the rat brain was performed using a custom-programmed segmented RF spoiled gradient echo (GRE) centric phase encode readout pulse sequence with a frequency selective continuous wave saturation preparation pulse. The sequence parameters were: field of view  = 35×35 mm^2^, slice thickness  = 2 mm, flip angle  = 15 degree, GRE readout TR  = 6.2 ms, TE  = 2.9 ms, matrix size  = 128×128, average  = 4 (voxel size ∼0.15 µl). CEST images were collected using a 1 second saturation pulse at peak B_1_ of 250 Hz for the frequencies (2.4, 2.6, 2.8, 3, 3.2, 3.4, 3.6, −2.4, −2.6, −2.8, −3, −3.2, −3.4, −3.6 ppm) from bulk water. For every 8 s one saturation pulse and 128 acquisition segments were applied.

For magnetization transfer ratio (MTR) mapping, same brain slices were imaged with saturation frequency set at 20 ppm with 250 Hz saturation power and 1 s saturation duration. Images with saturation frequency set at 100 ppm were also collected and considered as magnetization off image.

### Radiofrequency field inhomogeneity (B_1_) and static magnetic field inhomogeneity (B_0_) maps

B_0_ and B_1_ inhomogeneities in the brain slice were corrected using B_0_ and B_1_ maps generated from the same slice. Using the gradient echo images collected at different TE  = 3.0, 3.5 and 4.0 ms the B_0_ map was derived by linearly fitting the accumulated pixel phase Δθ_0_ following phase unwrapping against the echo time differences (Δ*TE*).
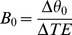
(1)


For B_1_ map, two images were obtained using preparation square pulses with flip angles *I*_1_ = 30^o^ and *I*_2_ = 60^o^ (pulse duration τ = 1 ms) followed by a spoiler gradient. Flip angle (*I*) maps were generated by solving Equation [2],
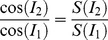
(2)where S(*I*_1_) and S(*I*_2_) denote pixel signals in an image with preparation flip angle *I*_1_ and *I*_2_ respectively. Using the relation 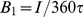, the B_1_ map was generated.

### Proton MRS

Single voxel spectra (SVS) were acquired with point resolved spectroscopy (PRESS) using a vendor (Varian) provided pulse sequence with the following parameters: voxel size  = 3.5 mm×3 mm×2 mm (Voxel volume 21 µL), spectral width  = 4 kHz, number of points  = 4006, averages  = 128, TE  = 14 ms, and TR  = 3 s. Voxel shimming was performed to obtain localized water line width values of ≤18 Hz (<0.05 ppm). Water suppression was achieved using the variable pulse power and optimized relaxation delays method (VAPOR). An unsuppressed water spectrum was also acquired using the same parameters for normalization. Both water suppressed and water reference spectra were analyzed using LC-model [21] and concentrations of glutamate (Glu) was obtained.

The total imaging time both for GluCEST and ^1^HMRS was ∼30 minutes.

### Image Processing

All image processing and data analysis were performed using software routines written in MATLAB (version 7.5, R2007b). Acquired images were corrected for B_0_ [19], [22] and used to generate GluCEST contrast map normally given as a relative change in % units using Equation [3]. 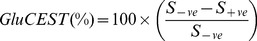
(3)where S_-ve_ and S_+ve_ are the B_0_ corrected MR signals at −3 ppm and +3 ppm from bulk water respectively. To account for and minimize the contribution from direct saturation and magnetization transfer effects, we used S_-ve_ instead of MR signal without any saturation (S_0_) for normalization. GluCEST contrast was further corrected for any B_1_ inhomogeneity [19], [22].

Similarly, MTR maps were computed using Equation [4]

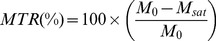
(4)


Where M_0_ and M_sat_ is the magnetization with a saturation pulse applied at 100 ppm and 20 ppm respectively.

## Results

The inter- and intra-animal coefficient of variation (CV) in measuring GluCEST is shown in Table 1. Less than 4% intra-animal GluCEST CV was observed from group 4. The inter-animal GluCEST CV was ∼13% which may reflect physiological variability in Glu concentration.

**Table 1:** Intra and inter- GluCEST coefficient of variance (CV).

Animals		Mean GluCEST (%)	SD	COV (%)
Group 4 reproducibility	Animal 1 (repetition = 5)	21.94	0.68	3.10
	Animal 2 (repetition = 5)	19.24	0.25	1.29
Inter-animal COV (N = 15)	All animals pre-injection	17.96	2.36	13.16

Table 1 shows the intra and inter- CV in measuring GluCEST contrast from rats brain.

**Figure 1:**
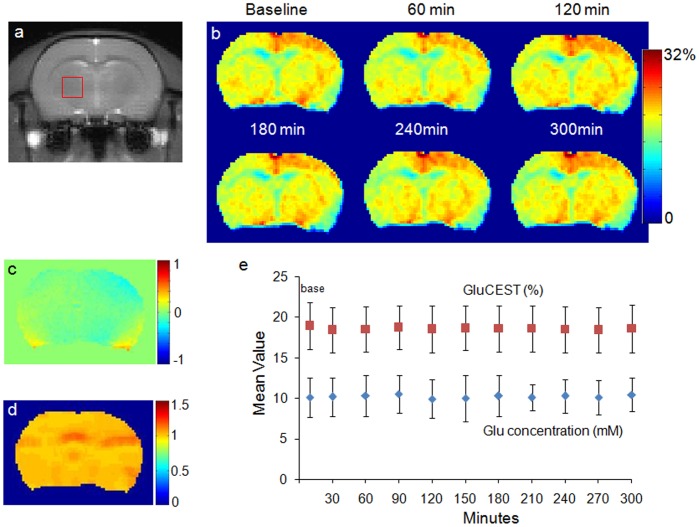
Chemical-exchange-saturation-transfer imaging of glutamate (GluCEST). (a) Anatomical brain image from one of the group 1 normal rat. (b) GluCEST maps of this rat brain pre and post Modafinil injection over time period of 5 hours. (c, d) B_0_ and B_1_ maps from corresponding brain slice as shown in (a). (e) graphs show no appreciable change in either mean GluCEST contrast or mean Glx concentration from ^1^HMRS over time period of 5 hours (n = 5) for the region of interest as shown in (a).

Figure 1a shows an anatomical brain image from a normal rat from group 1. The GluCEST maps at successive time points pre and post Modafinil administration over 5 hours are shown in Figure 1b. The corresponding B_0_ (Fig. 1c) and B_1_ (Fig 1d) maps show homogeneous field distribution (<20% variation) over the brain. The mean (SD) value of the GluCEST and Glu concentration from ^1^HMRS pre and post Modafinil injection in five rats at 30 minute intervals over the time period of 5 hours are shown in figure 1e. No appreciable change in either GluCEST contrast or Glu concentration was observed post Modafinil over the time period of 5 hours.

**Figure 2:**
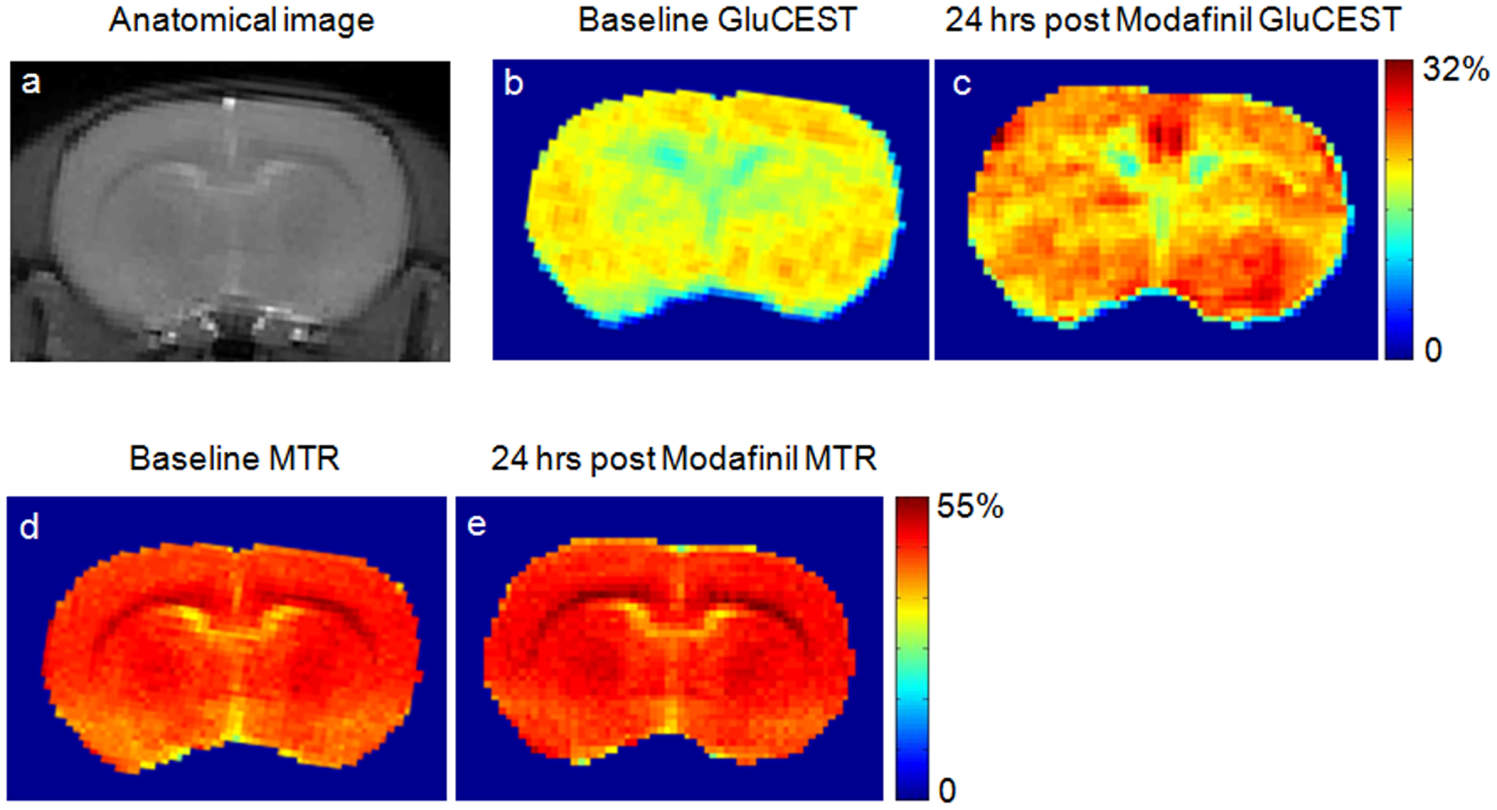
GluCEST map of a healthy rat brain from one of the group 2 animals pre and 24 hours post Modafinil injection. (a) Anatomical brain image from one of the group 2 normal rat. (b, c) GluCEST maps show an increase in GluCEST contrast 24 hours post Modafinil administration. (d, e) Magnetization transfer ratio (MTR) maps show no observable change in MTR contrast 24 hours post Modafinil administration.

Anatomical brain image from a normal rat from group 2 is shown in figure 2a. The corresponding GluCEST maps pre and 24 hours post Modafinil administration clearly show changes in GluCEST (Figures 2b and 2c) contrast. The other factor that may significantly contribute to the GluCEST is magnetization transfer effect from bound water pool. However, no significant change in the MTR contrast was observed before and 24 hours post Modafinil administration (Figures 2d and 2e).

**Figure 3:**
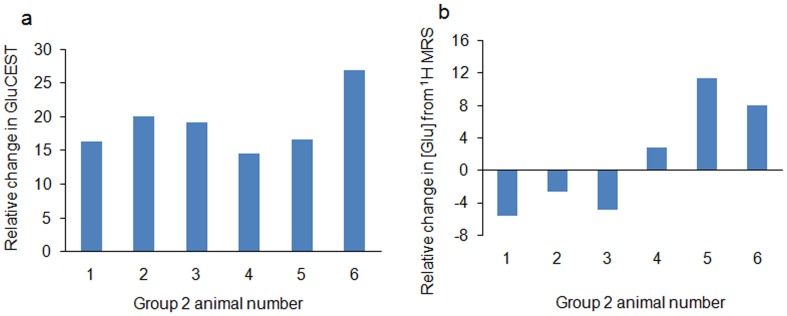
Relative change in GluCEST and Glx concentration from ^1^HMRS. Bar graphs show relative change in GluCEST contrast (a) and Glx concentration from ^1^HMRS (b) 24 hours post Modafinil administration for the group 2 rats (n = 6). While the change in GluCEST was positive in all rats, the change in Glx concentration was inconsistent.

Bar graphs in Figure 3 show the relative changes in GluCEST (Figure 3a) as well as Glu concentration from ^1^HMRS (Figure 3b) from group 2 rats pre and 24 hours post Modafinil administration.

For group 2 animals (n = 6), the mean value of GluCEST contrast and Glu concentration at baseline were 17.37±1.5% and 11.79+1.0 mM, respectively, while 24 hours post Modafinil administration these values were ∼21±1.6% and 11.96±1.1 mM. Student “t-test” was performed between pre Modafinil injection and 24 hours post Modafinil injection data from GluCEST and ^1^HMRS. The GluCEST contrast was significantly different (p<0.001) 24 hours post Modafinil administration whereas Glu concentration from ^1^HMRS was not significantly different (p∼0.30).

These results show that there is a difference in estimated Glu concentration changes and GluCEST changes.

For group 3 rats (n = 2) injected with the dose of 200 mg/kg Modafinil, no change either in GluCEST or Glu concentration was observed after 24 hours.

## Discussion

In the current study, for the first time we have quantified the Modafinil induced changes in Glu at high spatial resolution in rat brain using GluCEST method. No observable change in GluCEST contrast was observed up to 5 hours after Modafinil administration. These results are consistent with the results reported from liquid chromatography (HPLC) studies of rodents administered Modafinil [15]. However, 24 hours post Modafinil administration an average ∼19% increased GluCEST contrast was observed for a dose of 500 mg/kg.

While there are no previous studies of measuring glutamate changes 24 hours post Modafinil administration, there have been paradoxical sleep deprivation (PSD) studies in rats up to 24 hours [15]. In the PSD study, it was shown that up to 5 hours of PSD there were no change in Glu, while significant Glu changes (∼25%) were observed 24 hours PSD. Results from our study are consistent with these findings. However, the clear mechanisms leading to these increases in Glu concentration are still not known and require further studies.

As previously described, only about 75% of the observed GluCEST contrast is from glutamate in the imaging slice [19]. If there are significant macromolecular (proteins and lipids) changes associated with Modafinil administration, then the observed GluCEST changes cannot be attributed solely to glutamate level changes. The changes in macromolecular concentration can be observed by quantifying the MTR contrast. Our MTR contrast observations in this study 24 hours post Modafinil administration indicate no appreciable alteration in the macromolecular concentration, and therefore the observable increase in GluCEST contrast can be attributed predominantly to the increased Glu concentration. Furthermore, GluCEST maps show that the Modafinil effect appears to change glutamate levels rather homogeneously in the brain slice that is imaged in this study. Further studies are required to determine whether this trend is applicable throughout the brain.

The Glu changes from ^1^HMRS (Figure 3b) seem to be very inconsistent with some animals showing small increase and some animals showing no change and some animals even showing a small decrease. The most likely cause for this is the low signal-to-noise ratio (SNR) in the ^1^HMRS spectra in this study. Since, we were interested in looking at deep brain structures, a volume RF coil with homogeneous B_1_ was used in this study. The sensitivity of ^1^HMRS from a small voxel with such a coil invariably yields lower SNR.

We observed no significant change in either GluCEST and Glu concentration in group 3 rats injected with a lower dose of Modafinil (200 mg/kg). We suggest that this could be due to lack of or insufficient penetration of Modafinil into the brain. Since our aim in this manuscript was to show that glutamate concentration elevated by Modafinil can be imaged using GluCEST, we did not measure brain levels of Modafinil at different doses. Further, study with a large number of animals and observation for longer duration (>24 hours) post Modafinil administration is required to analyze dosage dependence.

The high intra-animal reproducibility (<4%) in measuring GluCEST suggests that it can be used to measure the changes in Glu concentration. A 13% inter-animal reproducibility most likely represents variation in the basal Glu concentration among rats.

In the current study, the higher sensitivity in measuring the GluCEST allows to depict smaller changes in the Glu concentration. The sensitivity can be further improved by optimizing the CEST pulse power and saturation duration.

Despite the numerous studies of oral Modafinil administration for both FDA and non-FDA approved, there are no reports of Modafinil effects on *in vivo* Glu levels in human subjects. Oral ingestion dosage levels of 200 mg to 400 mg/day of Modafinil is recommended for adult human subjects [23], [24], which corresponds to a chronic dosage of about 2 mg - 4 mg/Kg, and is significantly less than the acute one-time dose we used in the current study. Furthermore, the delivery of the drug in our study is with a water insoluble formulation injected via intraperitoneal route as opposed to oral administration in human studies. This difference may affect the bioavailability of the drug. Hence, it is rather difficult to compare the effects of Modafinil dose levels used in rat studies performed in this work with those used in humans. To assess the effect of chronic low dose levels of Modafinil injection as in human subjects, long term rat brain studies need to be performed with new protocols to be developed for Modafinil administration.

Our group has previously demonstrated that the GluCEST imaging can be performed in normal volunteers at 7T human scanner with high spatial resolution [19]. GluCEST method holds great promise as a tool to examine the Glutamatergic system in the pathophysiology and treatment of neurological, neuropsychiatric and substance dependence disorders.

In summary, GluCEST mapping allows regional cerebral Glu changes to be measured at high spatial resolution following Modafinil administration, and may provide a clinical biomarker of Modafinil effects that is useful for the management of patients with sleep, cognitive and substance dependence disorders.
